# Exploring probiotic awareness, perceptions, and practices in the Saudi population: a cross-sectional study

**DOI:** 10.7717/peerj.20490

**Published:** 2025-12-05

**Authors:** Basmah F. Alharbi

**Affiliations:** Department of Basic Health Sciences, College of Applied Medical Sciences, Qassim University, Buraydah, Saudi Arabia

**Keywords:** Probiotics, Knowledge, Perception, Practices, Saudi Arabia, Cross-sectional study, Adults, Epidemiology

## Abstract

**Background:**

Probiotics are composed of trillions of microorganisms, mainly located in the gastrointestinal tract (GIT), which play vital roles in digestion, immunity, and overall well-being. They help maintain gut health and prevent various gastrointestinal disorders. Despite the widespread use of probiotics in developed countries, such as North America and Western Europe, little is known about their use in Saudi Arabia. This study aimed to assess the knowledge, attitudes, and factors influencing probiotic awareness among the Saudi public to support healthier dietary choices.

**Materials:**

A cross-sectional study using a structured questionnaire titled “Public Knowledge and Perception of Probiotics” was conducted among Saudi residents aged 18 years and older from July to October 2024. With a type I error rate of 5% (α = 0.05) and 80% power, the required sample size was 269; ultimately, 286 individuals participated in the study. Data were analyzed using the Statistical Package for the Social Sciences version 27 (IBM).

**Results:**

The majority of the 286 participants were female (79%) and over 30 years old (54.9%). Awareness of probiotics was moderate (mean score: 5.2; 65%), with yogurt being the most recognized source (95%). Participants generally had positive perceptions (mean score: 3.74 ± 0.60), although barriers such as limited knowledge (56.2%) and high cost (3.28 ± 1.09) were reported. Probiotic use was noted in 54.5% of the respondents, with 71.2% preferring natural sources. Usage was significantly associated with demographic factors, including age ( $\chi^2$ = 15.251, *P* < 0.001), education level ( $\chi^2$ = 18.787, *P* < 0.001), marital status ( $\chi^2$ = 9.825, *P* = 0.002), and monthly income ( $\chi^2$ = 8.548, *P* = 0.014).

**Conclusion:**

While awareness and positive perceptions of probiotics are growing among the Saudi population, challenges such as knowledge gaps, affordability, and limited advertising persist. To promote probiotic use and improve public health outcomes, it is crucial to enhance public health education through schools and community programs, improve affordability *via* supportive health policies, and encourage greater involvement of healthcare professionals in providing probiotic-related guidance.

## Introduction

The human microbiota comprises approximately 10–100 trillion cells (Ursell et al., 2012), most of which reside in the gastrointestinal tract (GIT) (Amara & Shibl, 2015). The gut microbiota plays a key role in digestion, immune regulation, and intestinal development (Ley et al., 2006; Torrazza & Neu, 2011; Collado et al., 2009; Ivanov et al., 2008). An imbalance in this microbial community, known as dysbiosis, is linked to increased intestinal permeability, altered immune responses, and chronic inflammation, contributing to inflammatory bowel disease, irritable bowel syndrome, metabolic disorders, and neurological conditions. Common symptoms include abdominal pain, bloating, diarrhea, constipation, and increased susceptibility to infections (Kerry et al., 2018).

Probiotics are nonpathogenic live microorganisms—such as *Lactobacillus acidophilus, Lactobacillus rhamnosus GG, Bifidobacterium bifidum, Streptococcus thermophilus,* and *Saccharomyces boulardii*—that promote microbial balance, especially in the GIT (Williams, 2010). Their popularity as dietary supplements continues to rise due to their benefits for digestion and immunity, supported by major advancements in probiotic science since the early 20th century (Williams, 2010; Sanders et al., 2018). While earlier studies reported limited evidence of effectiveness in conditions such as bipolar disorder and certain skin diseases (D’Souza et al., 2002), recent findings suggest a possible gut–skin axis with benefits in acne, eczema, and psoriasis (Kim & Kim, 2023). Well-established therapeutic uses include the prevention of antibiotic-associated diarrhea (Boyle, Robins-Browne & Tang, 2006). Because probiotics are marketed as dietary supplements rather than medical drugs, they are not subjected to strict pre-marketing purity and safety regulations, which may result in variations in the actual content of commercial preparations (Theunissen et al., 2005).

The digestive system hosts numerous microorganisms that significantly influence the health of the gut (Pandey, Naik & Vakil, 2015; Gupta & Garg, 2009). The International Scientific Association for Probiotics and Prebiotics (ISAPP) defines probiotics as “live microorganisms that, when administered in adequate amounts, confer a health benefit on the host” (International Scientific Association for Probiotics and Prebiotics (ISAPP), 2025). Commonly used probiotic strains include *Lactobacillus*, *Bifidobacterium*, *Escherichia* *coli*, *Enterococcus*, *Bacillus*, and *Streptococcus*, along with some fungal species such as *Saccharomyces* (Gupta & Garg, 2009; Bermudez-Brito et al., 2012; McFarland, 2006). They are primarily derived from *the genera Lactobacillus, Bifidobacterium, and Enterococcus* and can be consumed through common foods such as yogurt, sauerkraut, bananas, leafy greens, onions, garlic, soybeans, and artichokes (Pandey, Naik & Vakil, 2015; Bermudez-Brito et al., 2012; McFarland, 2006). Probiotics help restore microbial balance disrupted by factors such as antibiotics, immunosuppressants, and radiation (Bermudez-Brito et al., 2012; McFarland, 2006). They support intestinal and immune functions and can reduce the risk of diarrhea (McFarland, 2006; Kechagia et al., 2013). Their effects are strain-specific, and while some evidence suggests a potential role in cancer prevention, the results remain inconclusive (McFarland, 2006). Although probiotics are generally safe, rare cases of sepsis, fungemia, and gastrointestinal (GI) ischemia have been reported in immunocompromised or critically ill individuals (Didari et al., 2013).

Given the growing body of evidence supporting the health benefits of probiotics, increasing public awareness is essential. Some developed nations, such as Canada (Bridgman et al., 2014), Australia (Braun et al., 2010), and the United States (Owens, Toone & Steed-Ivie, 2014), which have advanced healthcare systems, have a high level of probiotic supplement awareness, knowledge, and consumption. For instance, the estimated prevalence of probiotic consumption among adults in countries such as Canada, Australia, and the United States is approximately 30–50%, whereas the reported usage in developing nations, such as Nigeria and Jordan, remains below 20% (Bridgman et al., 2014; Braun et al., 2010; Owens, Toone & Steed-Ivie, 2014; Amarauche, 2015; Ayyash et al., 2021). However, this contrasts with some developing nations, such as Nigeria (Amarauche, 2015), Jordan (Ayyash et al., 2021), and the Arab Gulf countries (Faden et al., 2018). Recent studies from the United Arab Emirates and Qatar have also reported variable levels of public awareness and interest in probiotics (Alqaydi et al., 2024; Bendriss et al., 2020), while findings from healthcare professionals in Turkey demonstrate that greater microbiota awareness is associated with increased probiotic use (Cemiloğlu & Yılmaz, 2025), underscoring the need for updated data from the general population in Saudi Arabia (Faden et al., 2018; Rajab et al., 2023; Allah & Prarthana, 2019). Improving public knowledge may support healthier dietary practices among Saudi adults and their families, thereby enhancing overall well-being. We hypothesize that the Saudi population will demonstrate moderate knowledge and positive perceptions despite growing global interest in probiotics, with varied practices influenced by sociodemographic factors such as age, education, income, and marital status. Therefore, this study aimed to assess the knowledge, perceptions, and practices related to probiotics among Saudi adults.

## Materials and Methods

### Study design and setting

A community-based, descriptive, cross-sectional study was conducted. Eligibility criteria included being a Saudi resident aged 18 years or older and willing to voluntarily participate. Participants provided written informed consent after receiving a comprehensive explanation of the study objectives and subsequently completed the multiple-choice questionnaire in approximately 10–15 min. Participants were also informed that participation was voluntary and that all responses would be kept confidential. Participants were recruited through social media, particularly WhatsApp and X (formerly Twitter), using a snowball sampling method. This nonprobability sampling technique facilitated rapid, cost-effective recruitment across diverse demographic groups, including those less accessible through conventional means. The initial participants were encouraged to share the survey link to promote outreach and increase participation. The sample size calculation was performed using the G*Power, with parameters set at a significance level of 0.05, a power of 80%, and a medium effect size (w = 0.30). This calculation was based on our *a priori* hypothesis that the Saudi population’s knowledge and perceptions of probiotics would be moderate, with demographic characteristics varying in usage patterns. The proportion of participants exhibiting moderate knowledge of probiotics was on the main focus. These parameters match general methodological guidelines, including those from Green (1991) and VanVoorhis & Morgan (2007), and follow the “rules of thumb” often used in behavioral studies to find the minimum sample size for detecting medium-sized effects. Although the minimum sample size was calculated to be 269, a total of 286 valid responses were collected and included in the final analysis, with data collection occurring between July and October 2024. The Deanship of Graduate Research Studies and Scientific Research at Qassim University approved this study (Ethical approval number 24-91-20).

### The questionnaire

A structured questionnaire titled *“Public Knowledge and Perception of Probiotics”* was used for data collection (see Supplemental 1). The questionnaire included 27 items (Ayyash et al., 2021; Faden et al., 2018; Alqaydi et al., 2024) adapted from existing, peer-reviewed, validated instruments. The selection of questions was based on relevance, clarity, and content validity, in accordance with the study objectives. Participants completed an Arabic-language online version of the questionnaire, which facilitated comprehension among non-English speakers. The questionnaire was divided into four parts: the first part addressed demographic characteristics, and the second part assessed the participants’ knowledge of probiotics and sources of probiotic-related information (eight items using a binary scale of True and False questions). Knowledge responses were scored by assigning 1 point for each correct answer and 0 for incorrect answers, with a total score of 8 used to reflect overall awareness, with a Cronbach’s alpha of 0.5. A Cronbach’s alpha of ≥0.5 is considered acceptable for exploratory knowledge-based scales, where items measure different factual concepts rather than a single latent construct (Nunnally & Bernstein, 1994). The third part aimed to understand the general perception of probiotic usage (six items on a five-point Likert scale), and a mean score of 0.7 was calculated to represent the participants’ perceptions, with a Cronbach’s alpha of 0.7. A reliability coefficient of ≥0.7 is widely accepted as demonstrating good internal consistency for attitudinal and perception scales in health research (Nunnally & Bernstein, 1994). The final part addressed participants’ previous experience with probiotics and their future willingness to take them (four items, using multiple-choice questions), which were analyzed descriptively as categorical variables.

### Statistical analysis

Data were analyzed using the Statistical Package for the Social Sciences version 27 (IBM Corp., Armonk, NY, USA). Descriptive statistics (frequencies and percentages) were used to report categorical variables (*e.g*., age, gender, educational level, residence, marital status, monthly income, and occupational status). Responses related to probiotic knowledge were summarized as the percentage of correct answers, perceptions were presented as means and standard deviations for Likert scale items, and practices were reported using percentages and frequencies for multiple-choice answers.

In the knowledge section, the participants received one point for each correct answer and zero points for each incorrect answer. The total knowledge score was then calculated from 8.

Independent-sample t-tests and analysis of variance were used to examine differences in knowledge and perceptions of probiotic use based on demographic characteristics. The association between different probiotic use practices and demographic characteristics were assessed using chi-square test.

Additionally, the study followed the Strengthening the Reporting of Observational Studies in Epidemiology (STROBE) guidelines for reporting observational research.

## Results

### Demographics

A total of 286 participants were included in the study. Approximately half (54.9%) were aged ≤30 years, and 45.1% were aged >30 years. Most participants (79%) were female, whereas 21% were male. Regarding education level, 53.8% of the respondents held a diploma or bachelor’s degree, 28.7% had a high school education or less, and 17.5% were postgraduates. The distribution of residences showed that 55.2% were lived in the central region, 15.7% in the western, 11.5% in the southern, 10.5% in the eastern, and 7% in the northern regions. Regarding marital status, 52.4% were married and 47.6% were unmarried. As for monthly income, the majority (64.7%) earned less than 10,000 SR, 24.1% earned between 10,000 and 20,000 SR, and 11.2% earned more than 20,000 SR. Regarding occupational status, the majority (90.6%) were non-healthcare workers and 9.4% were healthcare workers, as shown in Table 1.

**Table 1:** Demographic characteristics (*N* = 286).

Variables	Categories	*N*	%
Age	30 years or less	129	45.1
	More than 30 years	157	54.9
Gender	Male	60	21.0
	Female	226	79.0
Educational level	High school or less	82	28.7
	Diploma or Bachelor	154	53.8
	Postgraduate	50	17.5
Residence	Central	158	55.2
	Northern	20	7.0
	Southern	33	11.5
	Eastern	30	10.5
	Western	45	15.7
Marital status	Married	150	52.4
	Unmarried	136	47.6
Monthly income	Less than 10,000 SR	185	64.7
	From 10,000 to 20,000 SR	69	24.1
	More than 20,000 SR	32	11.2
Occupational status	Healthcare worker	27	9.4
	Non-healthcare worker	259	90.6

**Note:**

Participant residence was recorded based on five geographic regions: Central (Riyadh, Qassim, Hail), Western (Makkah, Medina, Tabuk, Al-Baha), Northern (Al-Jouf, Northern Borders), Eastern (Eastern Province including Dammam), and Southern (Asir, Jazan, Najran).

### Knowledge, perception, and practices regarding probiotic use

The overall knowledge level regarding probiotics was 65%. Of the eight items, six had a correct response rate of >50%, ranging from 56% to 95%. The most correctly answered item was “Yogurt might contain a probiotic” (95%), followed by “Probiotics are not dead microorganisms” (79%), “Probiotics can be given to children” (71%), and “Milk might contain a probiotic” (70%). Additionally, 66% correctly identified that probiotics are not substances that make food taste sweeter, and 59% knew that they are not cleaning products used to kill bacteria on fruits and vegetables. However, only 45% of the respondents recognized that probiotics are not a type of vitamin, and 34% were aware that they can help prevent or cure viral infections. These results are presented in Table 2.

**Table 2:** Participants’ knowledge about probiotics.

No	Items	%
1	Probiotics are dead microorganisms, that when administered in adequate amount, confer a health benefit to the host*	79
2	Probiotics are substances that make food taste sweeter*	66
3	Probiotics are cleaning products to help kill bacteria on fruit and vegetables*	59
4	Probiotics are a type of vitamin to help health*	45
5	Probiotics can be used to prevent/cure viral infections	34
6	Milk might contain a probiotic	70
7	Yoghurt might contain a probiotic	95
8	Probiotics can be given to children	71
	Total mean (%)	5.2 ± 1.13 (65)

**Note:**

* False is the corrected answer.

Participants exhibited positive perception toward probiotic use, with a mean perception score of 3.74 ± 0.60 (Table 3). The item with the highest score was “There is a lack of advertising for probiotics and their benefits in Saudi Arabia,” with a mean score of 4.18 ± 0.90, followed by “Incorporating probiotics in our daily meals improves health and reduces illness,” with a mean score of 3.98 ± 0.97, followed by “Dietary sources of probiotics are better than pharmaceutical supplements,” with a mean score of 3.86 ± 1.02, followed by “There is a limited use of probiotics by people in Saudi Arabia,” with a mean score of 3.71 ± 0.89, followed by “Consuming probiotics in all forms is safe for health,” with a mean score of 3.44 ± 1.04, followed by “The high cost of probiotics discourages people from using them,” with a mean score of 3.28 ± 1.09.

**Table 3:** Participants’ perception of probiotic use.

No	Items	Mean ± SD
1	Consuming probiotics in all forms is safe for health	3.44 ± 1.04
2	Dietary sources of probiotics are better than pharmaceutical supplements	3.86 ± 1.02
3	Implementing probiotics in our daily meals improves health and reduces illness	3.98 ± 0.97
4	There’s a minor use of probiotics by people in Saudi Arabia	3.71 ± 0.89
5	There’s a lack of advertising probiotics and their benefits in Saudi Arabia	4.18 ± 0.90
6	The high cost of probiotics is one reason why people are reluctant to use probiotics	3.28 ± 1.09
	Total	3.74 ± 0.60

**Note:**

SD, standard deviation.

The results in Table 4 illustrate the use of probiotics. Approximately half of the participants (54.5%) had taken a probiotic; among them, 71.2% preferred probiotics from natural sources, 16% from pharmaceutical preparations/dietary supplements, and 12.8% had no preference (both can be used). The majority (73%) reported that they would continue using probiotics, 22.4% said they would not, and only 3.8% said they were unsure about continuing. The main reason for not using probiotics was a lack of knowledge about their benefits or uses (56.6%), followed by no perceived need (23.8%), fear of possible side effects (14.6%), and high cost (4.6%). Lastly, 0.8% did not believe in their effectiveness. Although the majority (53.1%) indicated that they would use probiotics in the future, 39.2% were uncertain about their use, and only 7.7% have no intention of using them. However, if healthcare providers recommended them, the intention to use increased; 83.8% stated they would, 13.8% were not sure, and only 2.3% said they would not use them.

**Table 4:** Participants’ practices of probiotics.

Variables	Categories	*N*	%
Have you ever taken a probiotic	Yes	156	54.5
	No	130	45.5
If you have used probiotics, which form of probiotic did you prefer to use** (*N* = 156)	Natural sources	111	71.2
	Pharmaceutical preparations\dietary supplements	25	16.0
	I have no preference (both can be used)	20	12.8
Are you willing to continue using probiotics** (*N* = 156)	Yes	115	73.7
	No	6	3.8
	Don’t know	35	22.4
If you did not use probiotics, what are the main reason for not using probiotics* (*N* =130)	Cost	6	4.6
	Possible side-effect	19	14.6
	I don’t believe in their effectiveness	1	0.8
	I don’t believe I need them	31	23.8
	I don’t know much about their benefits/uses	73	56.2
Are you willing to use probiotics in future* (*N* = 130)	Yes	69	53.1
	No	10	7.7
	Don’t Know	51	39.2
Would you use probiotics if they were recommended by your health care provider* (*N* = 130)	Yes	109	83.8
	No	3	2.3
	Don’t know	18	13.8

**Notes:**

* Percentage calculated at *N* = 130.

** Percentage calculated at *N* = 156.

### Differences in knowledge, perception, and probiotic practices due to demographic characteristics

The results indicate a significant difference in knowledge level based on educational attainment (F = 3.40, *P* = 0.035), with postgraduate participants demonstrating (M = 5.64 ± 1.38) higher knowledge levels than those with a high school education or less (M = 5.05 ± 1.40) (*P* = 0.036). A significant difference in knowledge was observed between probiotic users and nonusers (t = 2.50, *P* = 0.013). Participants who used probiotics reported higher knowledge scores (M = 5.38 ± 1.25) than nonusers (M = 4.99 ± 1.41) (Table 5). However, no significant differences were observed based on other demographic characteristics (age, gender, residence, marital status, monthly income, and occupational status). A significant difference in perception level was observed with respect to marital status (t = 3.235, *P* = 0.001), where married individuals (M = 3.85 ± 0.63) exhibited a higher positive attitude than their unmarried counterparts (M = 3.62 ± 0.55). There was also a significant difference in perception between probiotic users and non-users (t = 4.35, *P* = 0.001). Probiotic users had higher perception scores (M = 3.88 ± 0.57) than nonusers (M = 3.58 ± 0.60). Nonetheless, no significant differences were noted in other demographic characteristics (age, gender, educational level, residence, monthly income, and occupational status).

**Table 5:** Knowledge and perception levels according to demographic characteristics.

Variables		Knowledge	Perception
Age	30 years or less	5.11	3.67
	More than 30 years	5.29	3.80
	t (P)	−1.122 (0.263)	−1.854 (0.065)
Gender	Male	4.92	3.73
	Female	5.28	3.74
	t (P)	−1.897 (0.059)	−0.085 (0.932)
Educational level	High school or less	5.05^**b**^	3.65
	Diploma or Bachelor	5.15^**a,b**^	3.77
	Postgraduate	5.64^**a**^	3.80
	F (P)	3.40 (0.035)	1.245 (0.290)
Residence	Center	5.23	3.77
	North	5.00	3.77
	South	5.36	3.68
	East	4.77	3.78
	West	5.38	3.64
	F (P)	1.252 (0.289)	0.574 (0.681)
Marital status	Married	5.27	3.85
	Unmarried	5.14	3.62
	t (P)	0.802 (0.423)	3.235 (0.001)
Monthly income	Less than 10,000 SR	5.15	3.74
	From 10,000 to 20,000 SR	5.14	3.73
	More than 20,000 SR	5.69	3.80
	F (P)	2.359 (0.096)	0.157 (0.854)
Occupational status	Healthcare worker	5.59	3.95
	Non-healthcare worker	5.17	3.72
	t (P)	1.583 (0.115)	1.907 (0.057)

**Note:**

F statistics value of ANOVA, t statistics value of independent sample t-test, *Post hoc* test (Tukey), a, b different letter for significant between groups.

Table 6 indicates a significant association of probiotic use and age ( ${\chi^2}$ = 15.251, *P* < 0.001), where older people (>30 years) had more probiotic use than those aged 30 years, educational level ( ${\chi^2}$ = 18.787, *P* < 0.001), where post-graduation with more probiotic use than other educational levels, marital status ( ${\chi^2}$ = 9.825, *P* = 0.002), where married individuals reported greater use, and monthly income ( ${\chi^2}$ = 8.548, *P* = 0.014), where those with a monthly income of >20,000 SR had the highest probiotic use. However, no significant association was found between the preferred source of probiotics and demographic characteristics or between continuing probiotic use and demographic variables.

**Table 6:** The association between different practices of probiotic use and demographic characteristics (the users of probiotics).

		Ever taken a probiotic	The preferred source of probiotic			Continuing probiotic use
			Natural sources	Pharmaceutical preparations/ dietary supplements	Both	
		*N* (%)	*N* (%)	*N* (%)	*N* (%)	*N* (%)
Age	30 years and less	54 (41.9%)	35 (64.8%)	11 (20.4%)	8 (14.8%)	38 (70.4%)
	More than 30 years	102 (65%)	76 (74.5%)	14 (13.7%)	12 (11.8%)	77 (75.5%)
	${\chi^2}$ (*P*)	15.251 (<0.001)*	1.695 (0.428)			0.578 (0.746)
Gender	Male	32 (53.3%)	24 (75%)	3 (9.4%)	5 (15.6%)	28 (87.5%)
	Female	124 (54.9%)	87 (70.2%)	22 (17.7%)	15 (12.1%)	87 (70.2%)
	${\chi^2}$ (*P*)	0.045 (0.832)	1.442 (0.486)			4.357 (0.113)
Educational level	High school or less	31 (37.8%)	24 (77.4%)	3 (9.7%)	4 (12.9%)	19 (61.3%)
	Diploma or Bachelor	87 (56.5%)	59 (67.8%)	15 (17.2%)	13 (14.9%)	66 (75.9%)
	Postgraduate	38 (76%)	28 (73.7%)	7 (18.4%)	3 (7.9%)	30 (78.9%)
	${\chi^2}$ (*P*)	18.787 (<0.001)*	2.362 (0.669)			4.329 (0.363)
Residence	Center	88 (55.7%)	63 (71.6%)	16 (18.2%)	9 (10.2%)	70 (79.5%)
	North	14 (70%)	10 (71.4%)	2 (14.3%)	2 (14.3%)	11 (78.6%)
	South	20 (60.6%)	13 (65%)	3 (15%)	4 (20%)	13 (65%)
	East	11 (36.7%)	7 (63.6%)	2 (18.2%)	2 (18.2%)	7 (63.6%)
	West	23 (51.1%)	18 (78.3%)	2 (8.7%)	3 (13%)	14 (60.9%)
	${\chi^2}$ (*P*)	6.582 (0.160)	2.994 (0.935)			6.468 (0.595)
Marital status	Married	95 (63.3%)	69 (72.6%)	15 (15.8%)	11 (11.6%)	73 (76.8%)
	Unmarried	61 (44.9%)	42 (68.95)	10 (16.4%)	9 (14.8%)	42 (68.9%)
	${\chi^2}$ (*P*)	9.825 (0.002)*	0.375 (0.829)			1.263 (0.532)
Monthly income	Less than 10,000 SR	93 (50.3%)	66 (71%)	13 (14%)	14 (15.1%)	65 (69.9%)
	From 10,000 to 20,000 SR	38 (55.1%)	27 (71.1%)	9 (23.7%)	2 (5.3%)	31 (81.6%)
	More than 20,000 SR	25 (78.1%)	18 (72%)	3 (12%)	4 (16%)	19 (76.0%)
	${\chi^2}$ (*P*)	8.548 (0.014)*	4.142 (0.387)			3.188 (0.527)
Occupational status	Healthcare worker	18 (66.7%)	10 (55.6%)	6 (33.3%)	2 (11.1%)	17 (94.4%)
	Non-healthcare worker	138 (53.3%)	101 (73.2%)	19 (13.8%)	18 (13%)	98 (71.0%)
	${\chi^2}$ (*P*)	1.767 (0.184)	4.546 (0.103)			4.553 (0.103)

**Notes:**

${\chi^2}$ chi square test.

* *P* significant at 0.05.

Table 7 shows a significant association between the willingness to use probiotics in the future and region of residence ( ${\chi^2}$ = 18.187, *P* = 0.020), with those living in the western region showing the highest level of willingness, followed by those who lived in the central region. However, no significant association was found with other demographic characteristics. Regarding the likelihood of using probiotics in the future if recommended, there was a significant association with gender ( ${\chi^2}$ = 7.910, *P* = 0.019), where females were more likely to use probiotics in the future if recommended than those aged >30 years, and with monthly income ( ${\chi^2}$ = 18.554, *P* < 0.001), where those with a monthly income of 10,000–20,000 SR were the most likely to use probiotics in the future if recommended. However, no significant association was found with other demographic characteristics.

**Table 7:** The association between different practices of probiotic use and demographic characteristics (the non-probiotic user).

		Willingness to use probiotics in the future	Likely to use probiotics in the future if recommended
		*N* (%)	*N* (%)
Age	30 years and less	42 (56%)	66 (88%)
	More than 30 years	27 (49.1%)	43 (78.2%)
	${\chi^2}$ (*P*)	3.896 (0.143)	2.388 (0.303)
Gender	Male	13 (46.4%)	19 (67.9%)
	Female	56 (54.9%)	90 (88.2%)
	${\chi^2}$ (*P*)	5.203 (0.074)	7.910 (0.019)*
Educational level	High school or less	28 (54.9%)	43 (84.3%)
	Diploma or Bachelor	33 (49.3%)	57 (85.1%)
	Postgraduate	8 (66.7%)	9 (75%)
	${\chi^2}$ (*P*)	4.558 (0.336)	2.311 (0.679)
Residence	Center	42 (60%)	62 (88.6%)
	North	1 (16.7%)	4 (66.7%)
	South	2 (15.4%)	10 (76.9%)
	East	10 (52.6%)	13 (68.4%)
	West	14 (63.6%)	20 (90.39%)
	${\chi^2}$ (*P*)	18.187 (0.020)*	9.244 (0.322)
Marital status	Married	27 (49.1%)	46 (83.6%)
	Unmarried	42 (56%)	63 (84%)
	${\chi^2}$ (*P*)	3.889 (0.143)	0.816 (0.665)
Monthly income	Less than 10,000 SR	49 (53.3%)	79 (85.9%)
	From 10,000 to 20,000 SR	17 (54.8%)	28 (90.3%)
	More than 20,000 SR	3 (42.9%)	2 (28.6%)
	${\chi^2}$ (*P*)	1.485 (0.829)	18.554 (<0.001)*
Occupational status	Healthcare worker	6 (66.7%)	9 (100%)
	Non-healthcare worker	63 (52.1%)	100 (82.6%)
	${\chi^2}$ (*P*)	1.166 (0.558)	1.863 (0.394)

**Notes:**

${\chi^2}$ chi square test.

* *P* significant at 0.05.

## Discussion

The importance of probiotics in Saudi Arabia has recently gained recognition as awareness of their ability to help fight bacterial and viral infections has grown. This study builds on previous studies (Faden et al., 2018; Alqaydi et al., 2024; Bendriss et al., 2020; Cemiloğlu & Yılmaz, 2025; Rajab et al., 2023; Allah & Prarthana, 2019) and explores the broader context of probiotic use within the Saudi community. It encompasses various demographic levels, with a significant portion (71.3%) being well educated, while others hold high school certificates or lower qualifications, and a majority are aged ≥30. This research thoroughly investigates the knowledge, perceptions, and practices related to probiotics among the Saudi population, offering valuable insights to inform public health initiatives.

Public knowledge about probiotics was moderate (65%), with strong recognition of yogurt as a probiotic source (95%). Awareness also extended to their suitability for children (71%) and milk as a potential source (70%). Notably, 79% correctly identified that probiotics are living organisms, demonstrating a solid understanding of their biological nature. However, knowledge gaps persisted regarding their therapeutic role, particularly in viral immunity, where only 34% were aware of this benefit, consistent with findings from previous regional studies (Baud et al., 2020; Harper et al., 2021; Lopez‐Santamarina et al., 2021). Despite progress, probiotic misconceptions remain common. For instance, 34% of the participants believed that probiotics enhance the taste of food, while 41% associated them with cleaning products. These findings underscore the need for targeted educational campaigns to correct misunderstandings and foster accurate knowledge. No significant differences in knowledge were observed across age, gender, residence, marital status, income, or occupation. This observation aligns with prior findings in Jordan and Saudi Arabia (Ayyash et al., 2021; Faden et al., 2018), suggesting that although probiotics are familiar as foods, their scientific and clinical benefits remain less understood in Middle Eastern societies. The significant influence of education level on knowledge (F = 3.40, *P* = 0.035) supports evidence from health literacy research (Bayati et al., 2018), indicating that higher education enhances access to scientific information and improves the interpretation of health claims. In Saudi culture, health decision-making is often shaped by trust in informal sources, such as family, social media, and community norms, which may contribute to persistent misconceptions, such as perceiving probiotics as vitamins or unrelated to immune function. These findings highlight the need for structured health education initiatives—especially through primary healthcare providers and culturally relevant media platforms—to support an accurate public understanding of probiotics.

This study also revealed increasing awareness and favorable perceptions of probiotics among the Saudi population, reflected in a mean perception score of 3.74. This finding is comparable to those of other regional investigations (Ayyash et al., 2021; Faden et al., 2018). Participants recognized the health benefits of probiotics, particularly their role in supporting gut health and immunity, and showed a preference for natural sources over pharmaceutical supplements. These patterns are consistent with the findings in Jordan (Ayyash et al., 2021), suggesting that in Middle Eastern communities, probiotics are viewed primarily as part of traditional diets rather than as medical or commercial products. Nonetheless, misconceptions persist: 34% of respondents incorrectly associated probiotics with flavor enhancement, a finding similar to that in Nigeria (Amarauche, 2015). This gap between positive beliefs and accurate knowledge highlights limitations in public understanding and suggests that information about probiotics may not be effectively communicated through reliable channels. Although overall perceptions were positive, challenges such as limited knowledge (56.2%), high cost, and insufficient advertising were reported. These barriers have also been noted in studies from Australia and Canada (Bridgman et al., 2014; Braun et al., 2010), where awareness is high but accessibility remains a concern. In the Saudi context, the promotion of their scientifically supported health benefits remains limited, which may explain the gap between positive attitudes and actual product utilization, although probiotic products are becoming more available.

Probiotic use was more common among older, married, and individuals with higher education or higher income. This is consistent with prior research from the United States and Europe (Owens, Toone & Steed-Ivie, 2014), where education and socioeconomic status significantly influence probiotic consumption. Married participants demonstrated more favorable attitudes than unmarried individuals (t = 3.235, *P* = 0.001), likely reflecting household-level health decision-making. Older and married adults may also have increased interaction with healthcare providers and a greater focus on maintaining family health, which could encourage probiotic use. The other demographic factors were not significantly associated with perception scores. This suggests that health literacy and economic capacity may be more influential determinants than age or gender alone.

The findings highlight the essential role of education in enhancing public understanding and addressing persistent misconceptions. Providing accurate and consistent information through trusted healthcare providers and widely used media platforms may help improve informed decision-making. To engage diverse audiences, public health strategies should prioritize accessible, targeted education. Additionally, improving affordability and increasing scientifically based advertising could encourage the wider adoption of probiotics. Future research should examine how tailored educational interventions shape knowledge and behavior and assess the long-term effects of population-level probiotic consumption.

Probiotic practices among participants varied: 54.5% reported prior use, with 71.2% preferring natural sources over supplements (16%). These results are consistent with Jordanian data (Ayyash et al., 2021), where cultural norms favor natural dietary sources. This suggests that probiotics are still perceived primarily as food components rather than medical or commercial health products in Saudi Arabia. Among users, 73.7% expressed willingness to continue using them, similar to trends in countries such as Canada and Australia (Bridgman et al., 2014; Braun et al., 2010), where positive health outcomes reinforce informed usage. In contrast, non-users in Saudi Arabia reported barriers, including lack of knowledge (56.2%), concern about side effects (14.6%), and cost (4.6%). These findings highlight that uncertainty regarding efficacy, safety, and appropriate consumption may deter long-term use even when perceptions are favorable. These issues are less pronounced in countries with robust public awareness efforts (Bridgman et al., 2014; Braun et al., 2010).

Probiotic use was significantly associated with several demographic characteristics. Older participants (>30 years) were more likely to use probiotics (65%), reflecting U.S. findings that link age with preventive health behaviors (Owens, Toone & Steed-Ivie, 2014). Usage was highest among postgraduates (76%), consistent with Nigerian studies connecting education to improved dietary decisions (Braun et al., 2010). Furthermore, married individuals (63.3%) reported greater engagement, echoing Jordanian research emphasizing family-oriented health behaviors (Ayyash et al., 2021). This may indicate that individuals with greater household responsibility and life experience are more proactive in adopting health-promoting practices. The highest usage rates were seen in participants earning above 20,000 SAR (78.1%), paralleling trends in Australia and Canada (Bridgman et al., 2014; Braun et al., 2010), where income influences access. This relationship underscores how economic capacity may determine the ability to purchase probiotic products in Saudi Arabia, especially since they are often marketed as premium items. The willingness to adopt probiotics in the future was strongest among residents of the western region and those earning between 10,000 and 20,000 SAR. These groups may be more exposed to health promotion efforts and diverse food markets, which can increase both awareness and accessibility. When recommended by healthcare providers, participants were also more receptive to probiotics, an influence well documented in European studies (Kechagia et al., 2013). This emphasizes the essential role of healthcare professionals in guiding evidence-based probiotic use within the community.

Overall, these findings illustrate both globally consistent patterns and challenges specific to Saudi Arabia. While demographic trends in probiotic usage are similar to those seen in other countries, cultural beliefs, economic limitations, and gaps in health promotion remain significant. Saudi public health initiatives should adapt successful international strategies to the local context to enhance uptake. Key measures to improve probiotic adoption include increasing affordability and strengthening the role of healthcare providers, both of which are essential for promoting public health benefits.

In conclusion, this study provides a valuable contribution to the understanding of public awareness, attitudes, and usage patterns of probiotics across Saudi Arabia’s diverse regions. A validated questionnaire adapted for the local context was used, and a sample size exceeding the minimum threshold was achieved, enhancing the reliability of the results. This study examined the relationship between probiotic use and demographic variables, providing insights for targeted health interventions. However, it has limitations: its cross-sectional design prevents causal inference regarding education, income, and probiotic practices, and reliance on self-reported data may introduce recall or social desirability bias. The generalizability of the online snowball sampling method is limited, particularly for individuals with limited internet access or those residing in rural areas. Future research should focus on longitudinal designs to assess changes in probiotic awareness and behaviors over time and qualitative studies to explore motivations, beliefs, and misconceptions related to probiotic use in various subpopulations. Expanding the sample to include underrepresented and rural communities would enhance the generalizability of the findings. Intervention-based research is also recommended to evaluate health education programs targeting probiotic literacy and usage.

## Conclusions

This study highlights the growing public awareness and positive perceptions of probiotics among Saudi adults, particularly among females, older individuals, and those with higher education or income levels. Yogurt was identified as most widely recognized source, and natural forms were preferred. However, misconceptions, particularly about the role of probiotics in viral infections, and barriers such as high cost and lack of advertising still persist. Factors such as age, marital status, and education significantly influence probiotic use. To promote informed dietary choices and improve public health, implementing targeted educational campaigns led by policymakers, health educators, and healthcare providers, along with improved affordability, is essential. Future research should use longitudinal designs to investigate long-term probiotic use patterns, conduct cross-regional and age-based comparative analyses, and assess the impact of culturally tailored interventions.

## Supplemental Information

10.7717/peerj.20490/supp-1 Supplemental Information 1 Raw data.

10.7717/peerj.20490/supp-2 Supplemental Information 2 STROBE checklist.

10.7717/peerj.20490/supp-3 Supplemental Information 3 Questionnaire.

## Additional Information and Declarations

### Data Availability

The following information was supplied regarding data availability:

The raw data is available in the Supplemental Files.
