# Supplementary material for: Exploring probiotic awareness, perceptions, and practices in the Saudi population: a cross-sectional study
Source: PeerJ. 2025 Dec 5;13:e20490. doi: 10.7717/peerj.20490 (PMC12684406; doi:10.7717/peerj.20490)
Supplement: Supplemental Information 3 [file peerj-13-20490-s003.pdf]

Assessment of Knowledge and Perceptions towards probiotics: A Cross-sectional Study on the Saudi Public Population

Age group
- 20-25 years old
- 26-30 years old
- 31- 35 years old
- Over 36 years old

Gender
- Female
- Male

Completed education level
- High school or less than high school
- Diploma
- Bachelor
- Master/ PhD

Place of residence
- Center of Saudi Arabia
- South of Saudi Arabia
- North of Saudi Arabia
- East of Saudi Arabia
- West of Saudi Arabia

Marital status
- Married
- Unmarried (Single, Widowed, Divorced)

Personal monthly income
- <5000 Saudi riyal
- 5001–10000 Saudi riyal
- 10001–15000 Saudi riyal
- 15001–20000 Saudi riyal
- > 20000 Saudi riyal

Occupation
- University student
- Employee, working in the health field
- Employee, I do not work in the health field
- Retired
- Self-employment

Probiotics are dead microorganisms, that when administered in adequate amount, confer a health benefit to the host
- True
- False

Probiotics are substances that make food taste sweeter
- True
- False

Probiotics are cleaning products to help kill bacteria on fruit and vegetables

- True
- False

Probiotics are a type of vitamin to help health

- True
- False

Probiotics can be used to prevent/cure viral infections

- True
- False

Milk might contain a probiotic

- True
- False

Yoghurt might contain a probiotic

- True
- False

Probiotics can be given to children

- True
- False

Consuming probiotics in all forms is safe for health

- Strongly agree
- Agree
- Neutral
- Disagree
- Strongly disagree

Dietary sources of probiotics are better than pharmaceutical supplements

- Strongly agree
- Agree
- Neutral
- Disagree
- Strongly disagree

Implementing probiotics in our daily meals improves health and reduces illness

- Strongly agree
- Agree
- Neutral
- Disagree
- Strongly disagree

There`s a minor use of probiotics by people in Saudi Arabia

- Strongly agree
- Agree
- Neutral
- Disagree
- Strongly disagree

There`s a lack of advertising probiotics and their benefits in Saudi Arabia

- Strongly agree
- Agree
- Neutral
- Disagree
- Strongly disagree

The high cost of probiotics is one reason why people are reluctant to use probiotics

- Strongly agree
- Agree
- Neutral
- Disagree
- Strongly disagree

Have you ever taken a probiotic?

- Yes
- No

If you have used probiotics, which form of probiotic did you prefer to use?

- Natural sources
- Pharmaceutical preparations\ dietary supplements
- I have no preference (both can be used)

If you did not use probiotics, what are the main reason for not using probiotics?

- Cost
- Possible side-effect
- I don’t believe in their effectiveness
- I don’t believe I need them
- I don`t know much about their benefits/uses

Are you willing to use probiotics in future?

- Yes
- No
- I don`t know

Would you use probiotics if they were recommended by your health care provider?

- Yes
- No
- I don`t know
